# Local and Widespread Pressure Pain Hyperalgesia Is Not Side Specific in Females with Unilateral Neck Pain that Can Be Reproduced during Passive Neck Rotation

**DOI:** 10.3390/jcm8081246

**Published:** 2019-08-18

**Authors:** Fernando Piña-Pozo, Alberto Marcos Heredia-Rizo, Pascal Madeleine, Isabel Escobio-Prieto, Antonio Luque-Carrasco, Ángel Oliva-Pascual-Vaca

**Affiliations:** 1 Physiotherapy Department, University School Francisco Maldonado, University of Seville, 41640 Osuna, Spain; 2 Health Area of Osuna, Andalusian Health Service, 41640 Osuna, Spain; 3 Department of Physiotherapy, Faculty of Nursing, Physiotherapy and Podiatry, University of Seville, 41009 Seville, Spain; 4 Sport Sciences, Department of Health Science and Technology, School of Medicine, Aalborg University, 9100 Aalborg, Denmark

**Keywords:** cervical spine, idiopathic neck pain, musculoskeletal disorders, pain threshold, sensitization

## Abstract

Current evidence for widespread hyperalgesia in non-specific neck pain (NSNP) is unclear. It is currently recommended to group NSNP patients according to pain-provoking movements. The aim of this study was to investigate local and widespread pain sensitivity in females with unilateral NSNP that is reproducible during passive neck rotation compared with matched controls, and to compare the side specific effect of pain location on pressure pain sensitivity among females with unilateral NSNP. Thirty-six females with unilateral NSNP evoked during passive ipsilateral (*n* = 20) or contralateral (*n* = 16) rotation toward the painful side were compared with 20 controls. Participants reported their pain intensity at rest and during passive neck rotation and completed the Neck Disability Index. Pressure pain thresholds (PPTs) were assessed bilaterally over the anterior scalene; the sternocleidomastoid; the levator scapulae; lateral to the spinous process of C6; the median, ulnar, and radial nerves; and the tibialis anterior. The ANOVA revealed lower PPTs in females with unilateral NSNP compared with the controls (all at *p* < 0.001), but no differences were found between the sides, nor was there any Group × side interaction. Among females with NSNP, those with higher pain intensity during ipsilateral rotation toward the painful side showed lower PPTs over the anterior scalene, median nerve, ulnar nerve, and tibialis anterior (all, *p* < 0.05) than females with higher pain intensity during contralateral rotation toward the painful side. These findings demonstrated bilateral local and widespread pressure pain hyperalgesia in females with unilateral NSNP that was reproducible during passive neck rotation compared with controls. There was no side specific effect of pain location on PPTs among females with unilateral NSNP.

## 1. Introduction

Approximately 40% of the adult population suffers annually from neck pain [1]. Neck pain represents, along with lower back pain, the leading cause for years lived with disability worldwide [2] and poses a considerable financial burden [3]. Non-specific neck pain (NSNP), defined as pain in the cervical region without an identifiable pathoanatomical cause and reproducible with repeated neck movements or postures [4], is the most common clinical type of pain.

Measurement of the pressure pain threshold (PPT) is of increasing value in clinical practice [5] and is commonly used to assess local and widespread pressure pain hyperalgesia in NSNP [5,6]. Assessing the mechanical sensitivity of deep structures by using PPT may help to discriminate between people with and without neck pain [7] and to classify clinically meaningful subgroups of NSNP patients [5]. It may also provide prognostic value for the chronicity of neck pain occurring after trauma [8] and help stratify responders versus non-responders to a given intervention [9]. Interestingly, PPT assessments have also revealed sex differences in a person’s response to experimentally induced pain [10]. Females usually report lower PPTs than males [11].

The current knowledge related to widespread hyperalgesia in NSNP is scarce and unclear [5,6]. Different pain responses have been observed in patients with NSNP depending on pain location (e.g., localized or widespread pain [12]), the duration of symptoms (e.g., acute or chronic pain [13]), and the laterality of pain (e.g., unilateral or bilateral pain [14]). The classification of patients into homogeneous subgroups with similar pain profiles may actually help improve treatment efficacy and provide a more thorough communication tool among professionals [15]. Therefore, studies with subgroups of NSNP patients based on pain location [16,17], the laterality of pain [18], or pressure pain sensitivity levels [5], are recommended [19]. Classification schemes commonly include evaluation of the relationship between movement and pain [20]. Thus, it has been recently suggested to cluster NSNP patients according to the movements that reproduce the experienced pain [21]. The current body of literature suggests that the muscles ipsilateral toward the painful side may be impaired [22].

Therefore, this study aimed to investigate local and widespread pressure pain sensitivity in females with unilateral NSNP that can be reproduced during passive neck rotation compared with matched healthy controls, and to compare the side specific effects of pain location on pressure pain sensitivity among females with unilateral NSNP. We hypothesised that females with NSNP would have lower PPT levels (than their matched controls), a side specific effect of pain location on pressure pain sensitivity in participants with unilateral NSNP. We also hypothesised increased sensitivity in females with higher pain intensity during ipsilateral compared to contralateral neck rotation toward the painful side.

## 2. Methods

### 2.1. Study Design

This study used a cross-sectional design to compare females with long-lasting unilateral NSNP that could be reproduced during passive neck rotation, with age and sex-matched controls from the same population-based cohort. The study protocol complied with the ethical guidelines of research involving human subjects, was conducted according to the Helsinki Declaration, and received ethical approval (number: 04/2014) from the Biomedical Research Ethics Committee of Andalusia, Spain.

### 2.2. Participants

From September 2014 to February 2018, females between 18 and 50 years, with or without unilateral NSNP, were recruited through referral by a primary care physician. Females were also recruited by responding to a local announcement in a public health centre in Southern Spain. NSNP was defined as pain not due to any known cause and with symptoms provoked by neck postures, movements, and/or palpation of the cervical muscles [23]. Using a body map chart, females with persistent unilateral pain that had lasted for at least three months [22], and was localised between the inferior edge of the spinous process of C2 and the scapular girdle [24], were recruited. Participants with NSNP had to rate their pain intensity during passive neck rotation higher than two in an 11-point Numeric Pain Rating Scale (NPRS) and have a score higher than five in the Neck Disability Index (NDI) [25]. The participants were distributed to one of the two NSNP groups based on the most painful passive neck rotation, e.g., ipsilateral or contralateral rotation toward the painful side that could reproduce the experienced pain. In parallel, a control group composed of females reporting no pain was recruited. The exclusion criteria were a concomitant diagnosis of a primary headache; a higher neck pain intensity in a neutral position than during passive neck rotation; a history of cervical spine or upper limb surgery; whiplash [26]; the diagnosis of fibromyalgia, or any neurologic, inflammatory or rheumatologic diseases; two or more positive signs of compressed nerves (changes in sensation, myotomal weakness in the arms, or alteration in deep tendon reflexes) [27]; radiological signs of neural compression or spinal stenosis [28]; having received manual therapy in the last month before data collection; analgesic and/or antiinflamatory treatment in the last 72 h [29,30]; diagnosis of anxiety or personality disorders; pregnancy; and involvement in litigation. Those in the control group were also excluded if they exhibited a history of recurrent pain or previous severe trauma to the neck, face or head.

### 2.3. Study Protocol

To ensure outcome assessor blinding [31], a single examiner collected all measurements and was blinded to the participants’ allocation groups. Demographic (age, height, body mass, and hand dominance) and clinical (side of neck pain, and duration of symptoms) data were collected during a single session lasting approximately one hour, in a room with controlled temperature and humidity.

### 2.4. Outcome Measures

With participants in a comfortable seated position, the therapist held the patient’s head and performed a passive and slow-speed end-rage neck rotation toward the painful and non-painful sides, with a 30 s rest between mobilizations to avoid increased sensitivity to movement-evoked pain [32]. Patients were advised to raise their thumb and stop the mobilization if unbearable pain occurred before the end-rage. Using an 11-point NPRS, with 0 denoting “no pain” and 10 denoting “maximal bearable pain”, participants were asked to report their current neck pain intensity in a neutral head position, and at the end-range, passive ipsilateral and contralateral neck rotation toward the painful side. Self-perceived neck disability was assessed with the Spanish version of the NDI [33]. The NDI is a valid and reliable tool [34], divided into 10 questions that use a 6-point Likert scale, with 0 representing “no disability” and 5 representing “great disability”. Higher scores in the NDI denote a higher level of disability.

Pressure pain thresholds, defined as the minimum necessary pressure force needed to evoke pain [35], were assessed using a digital pressure algometer, model FPX 25 (Wagner Instruments, CT, USA), using a 1 cm^2^ contact probe. The measured thresholds were converted to kPa. The pressure algometry over neck muscles has shown high reliability in healthy individuals (ICC 0.91 (95% CI 0.82–0.97)) [36] and in patients with chronic NSNP (ICC 0.78–0.93 (95% CI 0.53–0.97)) [37]. Pressure pain thresholds were measured bilaterally (e.g., painful and non-painful sides in females with unilateral NSNP and dominant and non-dominant sides in the matched controls) over the following neck sites: the anterior scalene muscle belly at a site located two cm above the clavicle, over the anterior part of the transverse process of C6 [38,39]; the levator scapulae muscle at the superior angle of the scapulae [39]; the insertional site of the sternocleidomastoid muscle over the mastoid process [38,39]; and at a site situated two cm lateral to the spinous process of C6. It is possible to stimulate this last location with the algometer layers of different underlying muscles (e.g., splenius capitis, semispinalis capitis, and cervicis [40]). Thus, this site was defined as the “posterior neck muscles”, according to previous guidelines [41]. Pressure pain thresholds were also measured over the brachial plexus nerve trunks. The pressure algometry of the upper limb peripheral nervous systems showed moderate to high reliability (ICC 0.73–0.99 (95% CI 0.33–0.99)) [42]. The median nerve was located in the cubital fossa, medial to the tendon of the biceps brachii; the ulnar nerve was identified between the medial epicondyle and the olecranon; and the radial nerve was palpated between the medial and lateral heads of the triceps brachii, over the medial to lower third of the humerus [43]. These sites are commonly used for manual palpation of the brachial plexus nerve trunks and were chosen for their accessibility and ease of location [44]. The tibialis anterior muscle belly was selected as a remote location often used to assess widespread hyperalgesia [43]. The assessments followed a random order, with a 30 s break between measurements to prevent temporal or spatial summation [45]. The average of three measurements was used for the statistical analysis. 

The level of sensitization was evaluated using the PPT Index, equal to the PPT score at each point/mean PPT score of the control group at the same spot × 100 [26]. The greater the value of the PPT index (%), the lower the degree of sensitization.

### 2.5. Statistical Analysis 

The software Tamaño de la Muestra^®^ (version 1.1, Universidad de Medicina, Madrid, Spain) was used for sample size calculation, taking into account an alpha level of 0.05 and a desired power of 80%. In order to detect a 20% clinically relevant difference in the comparison of PPT scores between groups with an estimated inter-individual coefficient of variation for PPT of 20%, at least 16 participants were required per group [26,46].

Statistical processing of the data was carried out using the PASW Advanced Statistics (SPSS Inc, Chicago, IL, USA) version 20.0. The normal distribution of the study variables was assessed with the Kolmogorov Smirnov test. A mixed-model analysis of variance (ANOVA) was used to evaluate the differences in PPTs and PPT indexes between groups (unilateral NSNP with a higher pain intensity during ipsilateral neck rotation toward the painful side, unilateral NSNP with higher pain intensity during contralateral neck rotation toward the painful side, and the control), and sides (painful vs. non-painful in females with NSNP, and dominant vs. non-dominant in the pain-free controls). Bonferroni post-hoc analysis was used for pairwise comparisons. The effect size was reported using eta squared (η^2^). The significance level was set at a *p* value < 0.05.

## 3. Results

**Figure 1:**
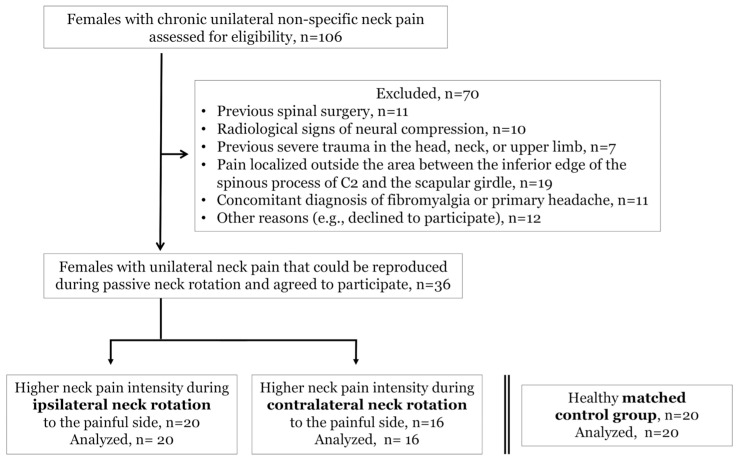
Flowchart diagram of the study participants.

One hundred and six females with persistent NSNP were assessed for eligibility. Finally, 36 females with unilateral NSNP that could be reproduced during passive neck rotation (20 of them reported higher pain intensity during ipsilateral rotation toward the painful side, and 16 reported higher pain intensity during contralateral rotation toward the painful side), as well as 20 pain free matched controls, volunteered to participate (Figure 1).

Table 1 lists the clinical and demographic characteristics of the participants. Females with NSNP reported higher pain intensity at rest and during passive neck rotation, as well as poorer levels of neck disability compared to the controls (*p* < 0.001). Among females with unilateral NSNP, those with higher pain intensity during contralateral neck rotation toward the painful side were younger than those with higher pain intensity during ipsilateral neck rotation toward the painful side (*p* = 0.031). Both subgroups of participants with NSNP reported similar scores in the NDI (15.1 ± 6.1 vs. 11.9 ± 4.8; *p* = 0.097). They also showed no differences in pain intensity at rest (*p* = 0.398), during the most painful neck rotation, whether ipsilateral (6.3 ± 1.6) or contralateral (5.6 ± 1.6) toward the painful side (*p* = 0.227), and during neck rotation toward the less painful side (3.4 ± 2.1 vs. 2.7 ± 2.4; *p* = 0.360).

**Figure 2:**
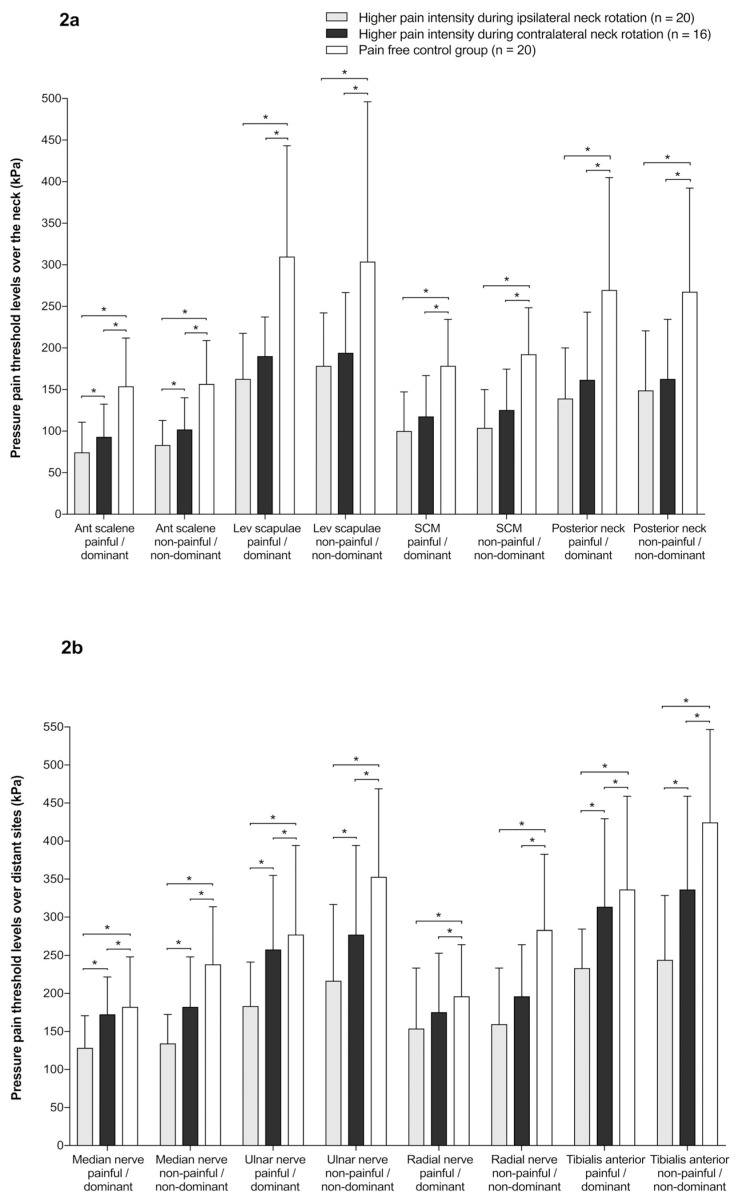
(**a**) Mean ± standard deviation (kPa) of the pressure pain threshold levels over the neck (the painful/dominant side and the non-painful/non-dominant side) in the study groups (*, *p* < 0.05). SCM, sternocleidomastoid muscle. (**b**) Mean ± standard deviation (kPa) of the pressure pain threshold levels (the painful/dominant side and the non-painful/non-dominant side) over distant sites in the study groups (*, *p* < 0.05).

For PPTs over the neck area, the ANOVA revealed significant differences between the groups at all sites: anterior scalene (F(2, 106) = 32.328; *p* < 0.001; η^2^ = 0.379)); levator scapulae (F(2, 106) = 23.397; *p* < 0.001; η^2^ = 0.306); sternocleidomastoid (F(2,106) = 28.464; *p* < 0.001; η^2^ = 0.349); and posterior neck muscles (F(2, 106) = 18.742; *p* < 0.001; η^2^ = 0.261) (Figure 2a). However, there were no significant differences between sides and no Group × Side interaction. For PPTs at distal sites, there were also significant differences between groups at the median (F(2, 106) = 41.724; *p* < 0.001; η^2^ = 0.440), ulnar (F(2, 106) = 25.480; *p* < 0.001; η^2^ = 0.325), and radial nerves (F(2, 106) = 28.247; *p* < 0.001; η^2^ = 0.348), as well as over the tibialis anterior muscle (F(2, 106) = 27.256; *p* < 0.001; η^2^ = 0.340), but, again, no differences between sides and no Group × Side interaction were found (Figure 2b).

**Figure 3:**
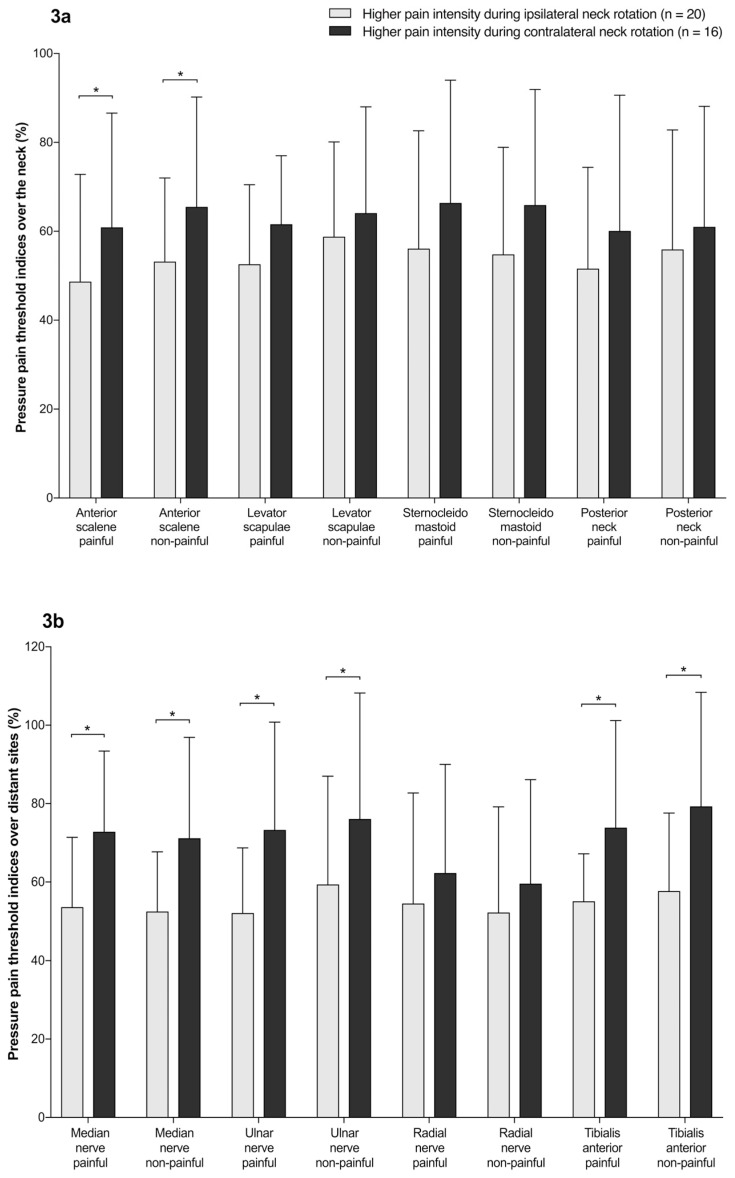
(**a**) Mean ± standard deviation (%) of the pressure pain threshold indices over the neck in females with unilateral non-specific neck pain that can be reproduced during passive neck rotation (*, *p* < 0.05). (**b**) Mean ± standard deviation (%) of the pressure pain threshold indices over distant sites in females with unilateral non-specific neck pain that can be reproduced during passive neck rotation (*, *p* < 0.05).

In participants with unilateral NSNP, the post-hoc comparisons revealed higher sensitization (lower PPT levels and PPT indices) in females with higher pain intensity during ipsilateral neck rotation toward the painful side compared to those with higher pain intensity during contralateral neck rotation toward the painful side over the following locations: anterior scalene (F(1,68) = 4.926; *p* = 0.030; η^2^ = 0.068), median nerve (F(1,68) = 16.093; *p* < 0.001; η^2^ = 0.191), ulnar nerve (F(1,68) = 9.263; *p* = 0.003; η^2^ = 0.120), and tibialis anterior (F(1,68) = 14.458; *p* < 0.001; η^2^ = 0.175) (Figure 3a,b). There were no differences between sides (e.g., the painful vs. non-painful side) at any assessed site. For the PPT index, the cervical region (anterior scalene, levator scapulae, and posterior neck muscles) showed a greater degree of sensitization (the lower PPT indices) to the distal sites (the ulnar nerve and tibialis anterior).

## 4. Discussion

As hypothesized, females with unilateral NSNP showed bilaterally increased local and widespread pressure pain sensitivity compared to the matched controls. Contrary to our hypothesis, there was no side specific effect on pain location in females with unilateral NSNP (i.e., no differences in PPT levels or indices between the painful and non-painful sides). However, we observed, as hypothesized, an increased sensitivity in the subgroup of females with higher pain intensity during ipsilateral, compared to contralateral, neck rotation toward the painful side.

### 4.1. Pressure Pain Sensitivity in Females with and without Unilateral Non-Specific Neck Pain

In line with most of the previous research assessing musculoskeletal pain in NSNP [13,26,41,43,47,48,49,50,51,52,53,54], our findings showed increased sensitivity to pressure stimuli on the necks of females with NSNP compared to healthy controls. Local hyperalgesia is a common feature of individuals suffering from chronic NSNP [6], which may reflect peripheral and segmentally related spinal cord sensitization [26]. In our study, females with unilateral NSNP also had increased sensitivity to pressure stimuli at distant locations compared to the matched controls, indicating widespread hyperalgesia. The observed differences in pressure pain sensitivity over local and distant locations between females with and without NSNP were above the 20% threshold considered to be clinically relevant [46]. Similar to our findings, previous research has demonstrated hyperalgesia at a remote site (e.g., the tibialis anterior [13,41,49,54,55]), the thigh [48], and/or the brachial plexus nerve trunks [13,41,47], in NSNP patients compared to pain-free individuals. On the other hand, some studies reported no significantly lower PPTs at a distant region in participants with chronic NSNP compared to healthy controls [26,43,47,50,53], and concluded that there were a lack of signs of central sensitization in these patients. Therefore, there is mixed evidence related to widespread hyperalgesia in NSNP [5,6]. Thus, further research including well defined subgroups of NSNP patients is necessary [6].

There are some possible explanations to account for the differences between studies. First, Walton et al. [5] recently identified four distinct clusters in patients with NSNP according to PPTs at local and remote sites. The so-called “low–low cluster”, denoting hyperalgesia at both the neck and the tibialis anterior, represented the most common type in females, accounting for 75% of cases, but only accounting for 45% of males with NSNP. This distinction can be explained by sex differences in response to experimentally induced pain [10]. Second, the duration of health history conditions (e.g., continuous medication intake and comorbid musculoskeletal pain) may be a contributing factor to widespread pressure pain hypersensitivity in this population [56]. Moreover, the duration of pain is suggested to be the responsible mechanism for the transition from acute localized pain to chronic widespread pain [57]. In our sample, the median duration of pain in females with NSNP was 60 months, supporting the conclusion that the duration of pain can contribute to widespread hyperalgesia. Third, widespread hyperalgesia in NSNP patients may be related to varying levels of self-perceived neck disability [55,58]. In fact, differences in the PPT at the tibialis anterior were observed between patients with mild and severe disability according to the NDI [30]. Fourth, increased sensitivity in NSNP may depend on the type of painful stimuli that is used (pressure, thermal, or electrical) [48,59]. Finally, patients with unilateral or bilateral NSNP show different sensory pain responses [14]. Interestingly, most previous studies either did not report the laterality of neck symptoms in their participants [41,43,47,49,50,51,53,54,55] or recruited only patients with bilateral pain [13,26]. To date, there has been only a single trial assessing PPTs in patients with unilateral NSNP [48]. This latter study included 18 participants, but only six of them were females, which makes a comparison between studies difficult.

Overall, the mechanisms underlying widespread pain in NSNP are complex and poorly understood. Widespread hyperalgesia has been formerly described in chronic pain disorders that commonly present unilateral manifestation, such as carpal tunnel syndrome [29], and epycondilalgia [60], as well as in patients with chronic unilateral musculoskeletal pain (e.g., unilateral shoulder pain [61], and unilateral thumb carpometacarpal osteoarthritis) [62]. However, based on current evidence, widespread pressure pain hyperalgesia may not be a feature of chronic NSNP but rather seems to be present in a subgroup of NSNP patients [6].

### 4.2. Pressure Pain Sensitivity in Females with Unilateral Non-Specific Neck Pain

We found no differences in PPT levels between the painful and non-painful sides among females with unilateral NSNP either at the neck or distant sites, which denotes no spatial specificity for sensitivity to pressure stimuli. In line with our current findings, Chua et al. [48] observed no side-to-side differences for PPTs over the neck, head, or thigh in patients with unilateral NSNP. Among females with unilateral NSNP, we found lower PPTs over the anterior scalene, the median and ulnar nerves, and the tibialis anterior in participants with higher pain intensity during ipsilateral rotation toward the painful side compared to those with higher pain intensity during contralateral rotation toward the painful side. This may suggest the presence of different sensitization mechanisms in females suffering from different types of unilateral NSNP [13,60] and illustrates the importance of screening with pain-provoking movements [21]. The heterogeneity of patients under the term NSNP requires dividing them into clusters with similar clinical features. This division may help us reach more definite conclusions about the local and widespread sensitization in NSNP [6] and improve clinical outcomes [18]. The classification of patients into homogeneous subgroups should take into account the relationship between movement and pain [20]. Indeed, it is recommended to subgroup NSNP patients according to the presence or absence of movement associated pain and the type of movement where pain appears [21]. Neck rotation range of motion is associated with pain intensity in patients with neck pain [63], and NSNP patients show less variability of movement during rotation compared to the healthy controls [64]. Thus, we used pain during neck rotation as the main factor with which to group our sample participants. In previous research, clusters of patients with NSNP have been made based on pressure pain sensitivity responses [5], levels of self-perceived neck disability [30], movement restriction in side flexion and rotation [65], neck posture [66], or the extent of their radiating pain and neurological signs [67]. The differences in the factors used to sub-classify NSNP patients problematizes further comparison among studies. 

The present findings should be cautiously interpreted for several reasons. We only included female patients, even though females usually report more pain intensity than males [68]. The sample size was appropriate for methodological purposes but relatively small from a clinical perspective and had a slight difference between the neck pain groups. Only patients with localized unilateral NSNP that could be reproduced during passive neck rotation were recruited, so the generalizability of the results is limited. Patients who report pain during active neck rotation may represent a different clinical subgroup than those assessed in the present study. This should be investigated in further research. Although a 30 s rest was implemented between passive rotations toward the painful and non-painful sides, the exact time interval needed to prevent the temporal summation of pain between pain-evoked movements is uncertain and may have influenced the NPRS scores. Pressure algometry is a cheap, reliable, and widely used tool in clinical settings. However, locating the brachial plexus nerve trunks with the algometer probe using anatomic landmarks is difficult, and other deep tissues may be involved [44]. Thus, the use of ultrasound for image guidance is recommended. The assessment of myofascial trigger points was not conducted when PPT measures were collected. Hence, the presence of either active or latent myofascial trigger points may have confounded the group results. Participants with cervical radiculopathy were excluded based on their history, a physical examination, and imaging using radiographs [27,69]. Additional testing, such as electromyography, was not performed. Finally, psychosocial and comorbidity-related aspects were not controlled in the present study.

## 5. Conclusions

The present findings revealed bilateral local and widespread pressure pain hyperalgesia in females with chronic unilateral NSNP that can be reproduced during passive neck rotation, compared to the matched controls. Among females with unilateral NSNP, there was no side specific effect of pain location on pressure pain sensitivity. Females with higher pain intensity during ipsilateral neck rotation toward the painful side showed higher sensitization than those with higher pain intensity during contralateral neck rotation toward the painful side. These preliminary results suggest different underlining mechanisms in both clinical presentations of unilateral NSNP, depending on the pain-evoked movement, which may indicate the need for different treatment approaches.

## Figures and Tables

**Table 1:** Clinical and demographic characteristics of participants (mean ± standard deviation or in frequency percentages).

	Higher NSNP during Ipsilateral Rotation (*n* = 20)	Higher NSNP during Contralateral Rotation (*n* = 16)	Control Group (*n* = 20)	*p* Value
Mean age (years)	41.1 ± 9.9	33.9 ± 9.3	36.6 ± 11.1	0.104
Height (cm)	162.1 ± 5.6	161.2 ± 5.4	162.1 ± 5.5	0.854
Body Mass (kg/m^2^)	24.7 ± 3.5	23.2 ± 3.1	23.4 ± 3.2	0.297
Most painful side; right % (*n*)	30% (6)	50% (8)	N/A	0.320 *
Hand dominance; right % (*n*)	90% (18)	94% (15)	90% (18)	0.889
Pain duration (months)	24 (6–260) ^†^	72 (12–264) ^†^	N/A	0.262 *
Neck Disability Index (0–50)	15.1 ± 6.1	11.9 ± 4.8	0.7 ± 1.1	< 0.001
NPRS (neutral position)	1.5 ± 2.1	2.2 ± 2.3	N/A	0.398 *
NPRS (ipsilateral rotation)	6.3 ± 1.6	2.7 ± 2.4	N/A	< 0.001 *
NPRS (contralateral rotation)	3.4 ± 2.1	5.6 ± 1.6	N/A	0.001 *

* Indicates differences between subgroups of participants with NSNP; ^†^ Median and interquartile range. NSNP, Non-specific Neck Pain; NPRS, Numeric Pain Rating Scale.
